# Probable Depression Is Associated with Lower BMI Among Women on ART in Kinshasa, the Democratic Republic of Congo: A Cross-Sectional Study

**DOI:** 10.3390/nu17203230

**Published:** 2025-10-15

**Authors:** Annie Kavira Viranga, Ignace Balaw’a Kalonji Kamuna, Paola Mwanamoke Mbokoso, Celestin Nzanzu Mudogo, Pierre Akilimali Zalagile

**Affiliations:** 1 Department of Nutrition, Kinshasa School of Public Health, University of Kinshasa, Kinshasa P.O. Box 11850, Democratic Republic of the Congo; annieviranda@gmail.com; 2 Food Science, Nutrition and Dietetics Section, Higher Institute of Medical Techniques of Kinshasa, Kinshasa P.O. Box 774, Democratic Republic of the Congo; balowaignace@gmail.com (I.B.K.K.); graciosamwa@gmail.com (P.M.M.); 3 Department of Basic Sciences, Faculty of Medicine, University of Kinshasa, Kinshasa P.O. Box 11850, Democratic Republic of the Congo; celestin.mudogo@unikin.ac.cd; 4 Patrick Kayembe Research Center, Kinshasa School of Public Health, University of Kinshasa, Kinshasa P.O. Box 11850, Democratic Republic of the Congo; 5 National Public Health Institute, Kinshasa P.O. Box 3243, Democratic Republic of the Congo

**Keywords:** depression, nutritional status, ART, HIV-positive, Kinshasa

## Abstract

**Background**: Women living with HIV (WLHIV) in low-income urban settings face multiple intersecting nutritional risks from food insecurity, poor dietary quality, and mental health problems. We evaluated the prevalence of household food insecurity and inadequate dietary diversity, examining their associations with depressive symptoms, antiretroviral therapy (ART)-related factors, and body mass index (BMI) among WLHIV attending routine ART clinics in Kinshasa, The Democratic Republic of Congo. This study addresses critical gaps in understanding the interplay between mental health and nutrition in the context of HIV care, with significant implications for improving health outcomes among vulnerable populations. **Methods**: In this clinic-based cross-sectional study (February–April 2024), we enrolled 571 women on ART in Masina 2, Kinshasa. Household food insecurity was measured using the Household Food Insecurity Access Scale (HFIAS), dietary diversity was assessed using the Minimum Dietary Diversity for Women (MDD_W; inadequate ≤ 5 food groups in 24 h), and probable depression was assessed using the Hopkins Symptom Checklist-10 (HSCL-10), which is a validated screening tool. We obtained baseline BMIs from clinic records at ART induction, which we measured again upon survey completion. We used analysis of covariance (ANCOVA) to model follow-up BMI, adjusting for baseline values, age, ART duration, self-reported adherence, household food insecurity, dietary diversity, and probable depression. Sensitivity analyses included change-score and mixed-effects models. **Results**: The prevalence of any household food insecurity was high (75%; 95% CI:71.5–78.6), with 57.6% (95% CI:53.5–61.6) of the participants experiencing inadequate dietary diversity (MDD_W < 5). Furthermore, forty-two per cent (95% CI:38.4–46.5) experienced depressive symptoms and sixty-eight percent (95% CI: 64.4–72.0) adhered to antiretroviral therapy (ART). The mean MDD_W was 4.3, with a low consumption rate of animal-source foods. Baseline BMI was associated with follow-up values (adjusted β_unstandardized_, 0.48 kg/m^2^ per 1 kg/m^2^ baseline, 95% CI 0.38–0.59; *p* < 0.001). Probable depression was independently associated with a lower follow-up BMI (adjusted β_unstandardized_, −0.99 kg/m^2^; 95% CI −1.72 to −0.26; *p* = 0.008). Time since ART initiation showed a slight positive association with BMI (adjusted β_unstandardized_, 0.10 kg/m^2^ per year). Self-reported ART adherence, household food insecurity, and dietary diversity were not independently associated with follow-up BMI in fully adjusted models. The interaction between age and probable depression did not suggest heterogeneity between age groups (*p* = 0.503). **Conclusions**: In our cohort, food insecurity and poor dietary diversity were widespread but did not significantly correlate with BMI, while probable depression, a potentially modifiable factor, was independently associated with lower BMI after accounting for baseline nutritional status. These findings highlight the need for HIV care programs integrating mental health screening and services with nutrition-sensitive interventions to support recovery and long-term health among WLHIV.

## 1. Introduction

Despite global progress in expanding access to antiretroviral therapy (ART), the nutritional and mental health needs of women living with HIV (WLHIV) in urban sub-Saharan Africa (SSA) remain inadequately documented. Research indicates that food insecurity prevents treatment adherence and ART effectiveness, which heightens the risk of disease progression and death [1]. Additionally, depressive symptoms are very common among WLHIV and have been associated with poor treatment adherence and a low quality of life [2]. Contexts with limited resources and interconnected challenges prevent the progress achieved by organizations like the World Health Organization (WHO) [3].

Malnutrition is a common and serious comorbidity in people living with HIV (PLHIV). It is linked to faster disease progression, higher morbidity, and increased mortality [3,4]. Recent studies emphasize the high prevalence of food insecurity and malnutrition, despite widespread ART use, especially among vulnerable groups like women [4]. This issue is worsened by unbalanced diets and a lack of necessary micronutrient diversity, which directly impacts overall health [5].

At the same time, mental health disorders, especially depression, are disproportionately high among PLHIV [1]. Depression is not only a risk factor for acquiring HIV, but also a common complication of the disease itself, as well as its treatment. Untreated depression can have severe consequences, affecting ART adherence and retention in care, which undermines viral suppression and clinical outcomes [6].

In clinical management, an often-overlooked crucial link is the complex interaction between depression and nutritional status. Depression can directly impair nutritional health by causing a loss of appetite, decreased food intake, and poor dietary choices. This vicious cycle of a weakened mental state and chronic malnutrition forms a major barrier to nutritional recovery and physical resilience [1]. Although studies have documented this connection in various contexts, data remains limited and fragmented, especially for specific urban populations in SSA.

To date, few researchers have thoroughly examined the relationship between depression and nutritional outcomes among HIV-positive women receiving ART.

This study aims to evaluate how depressive symptoms are associated with changes in nutritional status (as represented by BMI), measured both at induction and time of survey, among clients receiving ART, after adjusting for adherence to ART, household food security, depressive symptoms, and dietary diversity. We hypothesize that more severe depressive symptoms are associated with poorer nutritional outcomes among women receiving ART, and that this relationship is partly mediated by reduced dietary diversity and increased household food insecurity. We acknowledge that this relationship is plausibly bidirectional (probable depression ↔ food insecurity/nutrition), as described in conceptual frameworks for HIV and food insecurity; we therefore classified the observed associations as hypothesis-generating. More specifically, we tested whether depressive symptoms are independently associated with BMI measured at time of the survey after adjusting for baseline BMI, household food insecurity (HFIAS), dietary diversity (MDD_W), ART adherence, and sociodemographic covariates. Where causal language is used, we interpret the findings as associations requiring longitudinal or interventional confirmation.

## 2. Methodology

### 2.1. Study Design and Setting

This clinic-based cross-sectional study used historical BMI data recorded at ART initiation from the files of three clinics in the Masina 2 health zone of Kinshasa (Elonga Hospital Center, Lunda Health Center, and Lumière Health Center). We collected exposures and follow-up BMIs in a single cross-sectional survey; therefore, the study assessed associations rather than causal effects. Data were collected between 25 February and 30 April 2024. These facilities were chosen due to their large capacity to accommodate women undergoing ART, a treatment regimen that uses a combination of at least three antiretroviral drugs from at least two different classes to suppress the replication of HIV in the body.

### 2.2. Participants and Sample Size

The sample size was determined to precisely assess the proportion of HIV-positive individuals experiencing food insecurity; national statistics from The Democratic Republic of Congo (DRC) indicated a proportion of 57% [7]. To calculate power, we employed the Kish–Leslie formula, utilizing a 5% precision level and 95% confidence interval (CI). The required minimum sample size was 377. A 10% contingency was included, with 583 patients invited to participate in the trial. The study involved women aged 18 years and older who had received antiretroviral therapy for at least 6 months and provided informed consent; a total of 571 women participated in and completed the survey (final *n* = 571).

### 2.3. Measuring Nutrition, Food Security, and Mental Health

#### 2.3.1. Anthropometry as the Primary Outcome

The primary outcome was nutritional status, which was assessed using BMI as a proxy. Weight was assessed using a calibrated digital scale (with a precision of 0.1 kg), and height was recorded using a stadiometer (with a precision of 0.1 cm). BMI was calculated as weight in kilograms divided by height in meters. Two BMI time points were utilized: BMI at ART induction (baseline; sourced from clinic records) and assessed during the survey (follow-up). The primary outcome was follow-up BMI, modeled continuously. Baseline BMI extraction and measurement standardization were performed, with information on baseline BMI at ART initiation retrospectively extracted from routine clinic records. Clinic anthropometry was performed by trained nursing staff using digital scales (measured while wearing light clothing) and a wall-mounted stadiometer for height; when multiple measurements were recorded close to the start of ART, we selected the measurement closest to the ART initiation date (within a 3-month window where available).

#### 2.3.2. Household Food Insecurity (Exposure/Covariate)

Food insecurity—the primary independent variable—was measured using the HFIAS developed by the USAID-funded Food and Nutrition Technical Assistance (FANTA) project [8]. The HFIAS is a validated instrument that has been shown to distinguish food-insecure from food-secure households across different cultural contexts. It is a compilation of nine questions designed to reflect universal domains of the experience of food insecurity, including (1) anxiety and uncertainty about household food supply; (2) insufficient quality (including food type variety and preferences); and (3) insufficient food intake and its physical consequences. For this study, results are presented categorically as (1) food-secure, (2) mildly food-insecure, (3) moderately food-insecure, or (4) severely food-insecure [1], and are further dichotomized into the categories food-insecure versus food-secure. Cronbach’s alpha was 0.83, demonstrating the scale’s good internal consistency for the sample.

#### 2.3.3. Dietary Diversity

Dietary intake was assessed using a 24 h recall method. Foods were classified into the ten Minimum Dietary Diversity for Women (MDD_W) food groups: (1) grains, white roots, tubers, and plantain; (2) pulses; (3) nuts and seeds; (4) dairy; (5) meat, poultry, and fish; (6) eggs; (7) dark green leafy vegetables; (8) other vitamin A-rich fruits and vegetables; (9) other vegetables; and (10) other fruits. A point was allocated for each group consumed, with a range of 0 to 10. The mean MDD_W score in the sample was 4.27, with a standard deviation of 1.31. Adequate dietary diversity is defined as MDD_W ≥ 5, while inadequate diversity is indicated by MDD_W < 5, serving as a binary indicator. The cumulative MDD-W scores were categorized into variables: adequate dietary diversity (consuming five or more food groups) and inadequate dietary diversity or dietary monotony (consuming less than five food groups) [7,9]. Dietary diversity was measured according to a single 24-h recall using the MDD_W food group classification. We acknowledge that a single day may not reflect habitual intake, increasing the possibility of random measurement error; this limitation was anticipated and is discussed below. Where possible, we interpret MDD_W as a population-level indicator of diversity, rather than a precise measurement of individual habitual intake.

#### 2.3.4. ART Adherence

Adherence was assessed through self-reporting of the number of days ART was missed in the preceding week. Non-adherence was defined as taking less than 95% of prescribed doses over the preceding 7 days (binary) [10]. Upon enrollment, ART-naïve participants received a DTG-containing regimen (i.e., DTG 50 mg with lamivudine 300 mg and tenofovir disoproxil fumarate 300 mg, once daily). For participants already receiving ART, we switched their previous (DTG-free) treatment to the DTG-containing regimen. Previous DTG-free treatment included stavudine (D4T) or zidovudine (AZT), combined with lamivudine (3TC) and either nevirapine (NVP) or efavirenz (EFV). As part of a standard follow-up, measurements of participants’ CD4 cell and complete blood counts were scheduled every three months. Participants attended health facilities every month for ARV refills [11].

#### 2.3.5. Depressive Symptoms

An assessment was conducted using the Hopkins Symptom Checklist-10 (HSCL-10) [12]. This instrument is recognized as a valid clinical tool for identifying probable depression within primary healthcare settings [13] and in various research contexts [14,15]. Additionally, it has been utilized in comparable studies in Uganda [15]. In the current study, participants were asked to indicate the extent to which each symptom applied using a four-point scale ranging from “not at all” (0) to “extremely” (3). The mean score for the 10 items—each rated on a scale from 0 to 3—was calculated by dividing the total score by 10. A cutoff of ≥1.6 was established to signify probable depressive symptoms [12]. Cronbach’s alpha for the sample was 0.94.

#### 2.3.6. Additional Covariates

Other covariates considered in this study included sociodemographic characteristics such as age (in years), education level, marital status, household size, and household wealth index. This index was utilized through principal component analysis [16], which generated an index based on various household assets, including a radio, tape recorder, television set, bicycle, hand torch, and horse or donkey cart. Additionally, housing conditions were assessed based on roof material, number of rooms, wall type, windows, latrine availability and type, and ownership of domestic animals. Participants in the study were ranked according to their wealth index score and subsequently categorized into tertiles, ranging from lowest (first tertile) to highest (third tertile). The third tertile reflects a higher socioeconomic position (SEP). Behavioral covariates included alcohol and tobacco use, with few missing values.

### 2.4. Data Collection Procedures and Quality Control

Participants were interviewed during routine visits to the treatment center from 25 February to 30 April 2024. An interviewer-administered questionnaire was designed based on the existing literature and subsequently used for data collection [17,18]. It was translated into French and Lingala and piloted with a sample of 20 respondents (not included in the final analysis). The interviews were conducted in either of these languages, which are those most commonly used in Kinshasa. The interviewers attended two training sessions covering 24 h recall probing, HFIAS/MDD_W/HSCL-10 administration, anthropometry, and ethical procedures. Regarding nutrition data processing, mixed dishes were disaggregated into constituent food groups using local recipes, while a presence/absence approach was used for MDD_W scoring. Scales and stadiometers were calibrated regularly, and data entry and removal followed pre-specified protocols.

### 2.5. Statistical Analysis

Data analysis was conducted using Stata version STATA 17 (Stata Corp, College Station, TX, USA). Univariate analysis was conducted to provide descriptive statistics for age and BMI, including means and standard deviations for continuous variables and proportions with 95% confidence intervals for categorical variables. Counts of missing data items are given as follows (Table S1): out of 571 participants, 32 had missing baseline BMI information, 13 had missing follow-up BMI information, 3 lacked adherence information regarding ART, and 1 had missing data for the wealth index and food security. Baseline and follow-up BMIs were analyzed among groups based on food security, depressive symptoms, ART adherence, and dietary diversification utilizing the *t*-test. We visualized BMI distribution using a histogram (Figure S1).

We used analysis of covariance (ANCOVA) to estimate associations between probable depression symptoms, nutritional exposure, and BMI at survey, adjusting for baseline values. Our primary model regressed follow-up BMI scores on centered baseline values, antiretroviral therapy adherence (binary), probable depression (binary), household food insecurity (binary), dietary diversity score (continuous), time since induction in years (continuous), age (continuous), and socioeconomic status (tertiles) using ordinary least squares. To evaluate the potential variation in the connection between depression and BMI across different ages, an interaction term between age and depression was incorporated into the multivariable model, and the log-likelihood ratio test was employed to determine its significance. If deemed significant at *p* < 0.05, distinct multivariate regression analyses were conducted by age group. Robust standard errors were used to account for heteroskedasticity when appropriate. Continuous predictors were centered to aid the interpretation. Model fit was assessed using R-squared, while multicollinearity was checked using variance inflation factors (VIFs). Because the MDD_W was originally validated for women aged 15–49, we conducted a sensitivity analysis restricted to participants in this age group and compared the coefficients and CIs to those of the full sample. As a complementary approach, we conducted change-score analyses (follow-up minus baseline BMI). To account for clustering by facility, we fitted a two-level mixed-effects linear regression with a random intercept for facility (REML estimation) and, as a robustness check, estimated ordinary least-squares models with robust standard errors. Model diagnostics included the assessment of linearity (component-plus-residual/avplots and lowess), residuals-versus-fitted and Q–Q plots, heteroskedasticity tests (Breusch–Pagan and White), variance inflation factors for multicollinearity, and influence diagnostics (Cook’s D); where appropriate, we report robust standard errors and sensitivity analyses, excluding influential observations. Missing data were handled via multiple imputations where applicable, and sensitivity analyses included change-score (follow-up minus baseline BMI) and mixed-effects models. Missing data were handled using multiple imputations by chained equations with m = 10 imputations. We combined estimates using Rubin’s rules and contrasted results with a complete-case analysis (Table S5). The imputation model included all variables from the analytical model (baseline and follow-up BMI, age, years since ART induction, probable depression, household food insecurity, dietary diversity score, adherence to antiretroviral therapy and socioeconomic status (tertiles)), plus auxiliary predictors related to missingness (Table S6). Convergence and plausibility were assessed via trace and over-imputation diagnostics. A significant level of *p* < 0.05 was set to ensure robust results.

The dataset was uploaded and is available at the following link: https://doi.org/10.17605/OSF.IO/35NJX.

### 2.6. Ethical Approval

This study was approved by the Ethics Committee of the Kinshasa School of Public Health, under reference ESP/CE/18/2024, on 8 February 2024. Fundamental ethical principles, including respect for the individual, beneficence, and justice, were strictly adhered to. Participants provided written informed consent prior to participation; data confidentiality and anonymity were maintained, and all archives—whether physical or digital—were accessed exclusively by the principal investigator in a secure space.

## 3. Results

The majority of the participants were aged 35–49 (39%). Of these, 55% possessed secondary education, and 83% refrained from alcohol consumption, stating consistent antiretroviral therapy adherence with a median duration of 4 years (Table 1).

### 3.1. Prevalence of Key Indicators Among Study Participants

Between 66.3% and 73.9% of participants reported experiencing each listed access-related food insecurity condition at least once during the recall period; the highest prevalence was worries about insufficient food (73.9%) and an inability to eat preferred foods (73.7%), while the lowest prevalence was identified for going a whole day and night without eating (66.3%). Frequency data indicate that relatively few households reported experiencing these conditions “often” (mostly 0–3.7% across items), with absolute “often” counts less, relative compared to “ever experienced” counts (Table 2).

**Figure 1:**
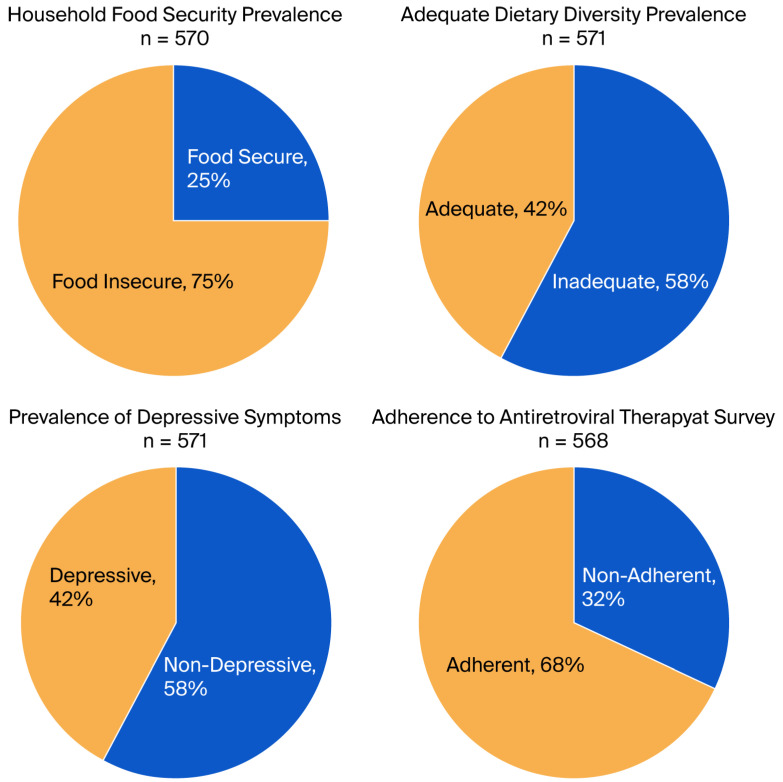
Prevalence of key indicators among study participants.

Table 3 presents the consumption frequency of various food groups among the studied population. A total of 558 participants reported consuming grains, white roots, tubers, and plantains, indicating a high prevalence at 97.7%. Dairy products followed closely with 30.3% (173 participants), while pulses—including beans, peas, and lentils—were consumed by 94.6% (540 participants). In contrast, consumption of meat, poultry, and fish was significantly lower, with only 68.7% (392 participants) reporting regular intake. Other notable findings include egg consumption at 8.8% (50 participants), and a modest intake of dark green leafy vegetables (66.5%, 380 participants), and various vegetables and fruits ranging respectively from 89.1% to 31.9%. Dietary diversity was assessed with a mean diversity score of 4.27 (±1.31). The results indicate that 57.6% of participants exhibited inadequate dietary diversity, while 42.4% achieved an adequate diversity level (Table 3 and Figure 1).

Our study identified significant vulnerability among the participants: 75% (95% CI:71.5–78.6) resided in food-insecure homes. Fifty-eight per cent of women (95% CI:53.5–61.6) had inadequate dietary diversity, while forty-two per cent (95% CI:38.4–46.5) experienced depressive symptoms. Sixty-eight percent (95% CI: 64.4–72.0) adhered to antiretroviral therapy (ART) (Figure 1).

Table S2 summarizes the stratified descriptive results, presenting the mean baseline and follow-up BMI categorized by the variables of probable depression, household food insecurity, ART adherence, and dietary diversification. Women exhibiting probable depressive symptoms had a lower mean follow-up BMI compared to those without such symptoms (22.3 vs. 24.1, *p* < 0.001). Supplementary Table S2 presents the comprehensive stratified counts and means. The overall mean dietary diversity score was 4.28, with no significant difference between the 15–49 age group and the 50 and older group (4.28 vs. 4.22; *p* = 0.573). The proportion of women exhibiting adequate diversity was comparable between the two groups (41.94 vs. 43.33; *p* = 0.755).

### 3.2. Changes in Body Mass Index Between Induction and Survey Day

**Figure 2:**
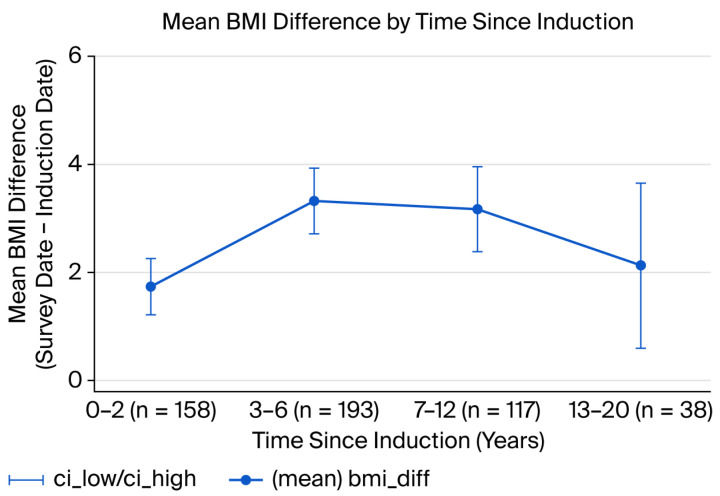
Mean BMI difference by years since induction (with 95% CI) (*n* = 506).

Changes were observed in post-induction BMI peaks at 3–6 years, with declining trends thereafter. The mean BMI difference (after–before) was greatest in the 3–6 year window (~3.4 units (95%CI: 2.69–3.94)), with slightly smaller but overlapping increases at 7–12 years (3.17; 95%CI: 2.35–3.99) and a reduced change by 13–20 years (2.12; 95%CI: 0.47–3.77). Confidence intervals indicate some uncertainty in the later periods, meaning that long-term trends should be interpreted with caution (Figure 2).

The prevalence of overweight and obesity escalated from baseline (12.5% overweight + 3.2% obese = 15.7%) to follow-up (22.0% overweight + 7.0% obese = 29.0%), though no significant variations in BMI distribution were observed based on adherence status at either baseline or follow-up (*p* = 0.397 at baseline, 0.441 at follow-up). Significant differences were, however, detected (baseline *p* = 0.013; follow-up *p* < 0.001) regarding probable depression status. Individuals exhibiting depressive symptoms demonstrated a greater baseline prevalence of underweight (35.9% versus 23.3%), and BMI distributions during follow-up significantly diverged from those of non-depressive participants. No significant relationships with BMI categories at baseline or follow-up were observed (*p* ≈ 0.806 at baseline, 0.420 at follow-up). At baseline, the results were not significant (*p* ≈ 0.317), but they became significant at follow-up (*p* = 0.004); poor dietary diversity was associated with a somewhat greater prevalence of overweight/obesity at follow-up compared to adequate diversity (Table S3). Figure S2 shows the changes in BMI, both overall and by key exposures.

In an ANCOVA model involving 503 women, baseline BMI was identified as the strongest associated factor of survey BMI (adjusted β_unstandardized_, 0.48, 95% CI 0.38–0.59; *p* < 0.001), indicating significant tracking of this variable over time. Participants with probable depression showed a lower adjusted BMI at the time of the survey compared to those without depressive symptoms (adjusted β_unstandardized_, −0.99 kg/m^2^, 95% CI −1.72 to −0.26; *p* = 0.008). Additionally, each extra year since induction was associated with a modest increase in BMI (adjusted β_unstandardized_, 0.10 kg/m^2^ per year; 95% CI 0.008–0.193; *p* = 0.034).

We found no evidence of association between ART adherence and follow-up BMI (adjusted β = −0.03, 95% CI = −0.73 to 0.66; *p* = 0.923), and no significant links were identified for household food insecurity or dietary diversity and BMI in fully adjusted models. The overall model explained 35.5% of the variation in follow-up BMI among participants (Table 4). The interaction between age and probable depression did not suggest heterogeneity between age groups (*p* = 0.503). A sensitivity analysis limited to individuals aged 15–49 years revealed a broad similarity when compared to estimates from the full sample (Table S4). For the multilevel (mixed-effects) model, the random-intercept mixed model produced similar fixed-effect estimates for baseline BMI (β = 0.476; 95% CI 0.411–0.540; *p* < 0.001) and centered time (β = 0.092; 95% CI 0.011–0.173; *p* = 0.027). The estimated between-facility variance (random intercept) was small (var(intercept) = 0.207 (standard deviation (SD) = 0.455); residual variance = 12.839 (SD = 3.583)). The intraclass correlation coefficient (ICC) was 0.016 (95% CI 0.0004–0.379), indicating that only ~1.6% of the total variance was attributable to facility-level clustering. A likelihood-ratio test of the random intercept vs. a linear model gave chibar2 = 0.86 (*p* = 0.176), consistent with a small and non-significant clustering effect. We therefore present the mixed-effects model as the primary clustered analysis and the cluster-robust OLS as a sensitivity analysis.

The coefficient estimates were similar, but the standard errors were larger when clustering was accounted for (For example, the centered baseline BMI robust standard error (SE) increased from 0.033 to 0.059 (the centered baseline BMI with clustering: β = 0.480; 95% CI 0.228–0.732; *p* = 0.015). The VIFs were low (mean VIF = 1.17; all VIFs ≤ 1.30), indicating no material multicollinearity. The Breusch–Pagan test did not refute homoskedasticity (χ^2^(1) = 2.15, *p* = 0.142), but White’s test indicated heteroskedasticity (χ^2^(48) = 165.39, *p* < 0.001), suggesting some non-constant variance, possibly related to higher-order/categorical interactions; thus, we present robust clustered SEs in sensitivity analyses. Residuals vs. fitted and Q–Q plots showed no major departures from linearity or normality, while component-plus-residual (av) plots for continuous predictors (baseline BMI, dietary diversity score, centered time, age) supported linear functional forms in the observed ranges. Influence diagnostics (Cook’s D) identified a small number of observations with elevated influence, with results robust to the exclusion of these observations (Figures S3 and S4, Table S5). When comparing complete-case and MICE approaches, both the complete case analysis and the multiple imputation by chained equations (MICE) approach reveal that baseline BMI is a significant associated factor of follow-up BMI, underscoring the critical role of initial weight status in determining long-term nutritional outcome (follow-up BMI) and identifying depression as a significant factor negatively impacting BMI changes (Table S6).

## 4. Discussion

In this cross-sectional analysis of 571 women living with HIV and taking ART in Masina 2, Kinshasa, we observed a high burden of household food insecurity (about 75%) and inadequate dietary diversity (57.6% with MDD_W < 5). More specifically, average MDD_W scores were low (mean 4.27 ± 1.31), and a high proportion of women consumed few animal-based foods, eggs, and dairy. At a population level, these patterns indicate an elevated risk of multiple micronutrient insufficiencies (e.g., iron, vitamin B12, zinc, and calcium). Baseline BMI strongly associated with BMI at the time of the survey (adjusted β 0.48, 95% CI 0.37–0.58; *p* < 0.001), while probable depression was independently associated with lower BMI at follow-up (adjusted difference −0.99 kg/m^2^, 95% CI −1.72 to −0.26; *p* = 0.008). Time since ART induction was associated with a small average annual increase in BMI (adjusted β 0.10 kg/m^2^ per year). We found no independent association between ART adherence, household food insecurity, or dietary diversity and follow-up BMI in fully adjusted models. These findings indicate that addressing depressive symptoms may significantly enhance nutritional recovery, supporting increasing evidence from SSA that mental health constitutes a critical determinant of HIV care outcomes.

Our findings align with growing evidence linking mental health symptoms, food insecurity, and poorer dietary quality among women in low-resource settings. Using household food security and diet quality metrics similar to those employed in this study, recent work in this region has documented associations between depressive symptoms and reduced dietary diversity or poorer nutritional status [19]. The high prevalence of food insecurity we observed is consistent with urban household surveys in Kinshasa and other Central African settings, which have reported frequently disrupted meal patterns and reliance on low-diversity diets in low-income households [8,20]. In the context of HIV, depression has been linked to reduced appetite, impaired caregiving and food procurement behaviors, and worse ART adherence, each of which contributes to declining nutritional status [21]. The strong tracking of BMI from baseline to follow-up is consistent with longitudinal studies showing persistent weight status among adults on ART; baseline nutritional status often sets a trajectory for later outcomes [22]. The negative association between depressive symptoms and BMI suggests that mental health may undermine nutritional status through reduced appetite, poor self-care, and lower access to resources due to functional impairment [23,24]. The modest increase in BMI with treatment time likely reflects recovery from HIV-related weight loss after ART initiation in the earlier years, with a plateau or attenuation over longer durations (note the peak change at 3–6 years in our sample) [25]. The increase in BMI following ART initiation likely reflects several processes. Early ART often leads to recovery from HIV-associated weight loss (return-to-health), improvements in appetite and energy, and reductions in opportunistic infections, all of which increase weight over months to years.

The lack of an independent association between self-reported ART adherence and BMI may reflect measurement limitations, such as competing drivers of weight change (e.g., baseline disease severity, comorbidities), or the fact that adherence in our sample (68% adherent) was insufficiently variable to detect an effect on BMI. In relation to this, adherence may influence viral suppression and clinical outcomes rather than directly associated with BMI. Without data on viral loads, CD4 and TB status, we cannot fully assess this pathway. In addition, some antiretroviral drug classes (notably some integrase strand transfer inhibitors) have been associated with weight gain, and changes in lifestyle or diet after engagement with care may contribute. In our cohort, self-reported 7-day ART adherence showed no association with BMI; this may reflect (i) an overall relatively high adherence with limited variability (a ceiling effect), (ii) the short recall window of the adherence measure vs. long-term weight change, (iii) measurement error in self-reported adherence, or (iv) that regimen type/duration (which we could not completely characterize for all participants)—rather than short-term adherence fluctuations—is a stronger driver of long-term weight change. The absence of ART regimen class and duration likely confounds BMI trends, such as the possible weight impacts of integrase inhibitors. This residual confounding may skew the depression–BMI relationship in either direction; our results should thus be regarded as correlational and hypothesis-generating. We note that more detailed prospective data on regimen changes, viral suppression, and body composition would be needed to disentangle these mechanisms.

Similarly, the absence of a direct association between household food insecurity or dietary diversity and BMI [26] in multivariable models may reflect the following: (a) BMI’s insensitivity to short-term or qualitative dietary deficits (micronutrient gaps can exist despite preserved BMI); (b) potential measurement errors or misclassification in the 24 h diet recall and HFIAS; or (c) mediation or confounding by depression for baseline nutritional status [27,28]. BMI is an imperfect indicator of nutritional quality and micronutrient status. Individuals may have normal BMI yet suffer from micronutrient insufficiencies, sarcopenia, or altered body composition, limitations particularly relevant in HIV populations. Our null findings for the relationship between dietary diversity and HFIAS with BMI likely reflect the insensitivity of BMI to short-term or qualitative dietary deficits, measurement errors during single 24 h recalls, and potential mediation/confounding by depression and baseline BMI. We therefore urge that this absence of associations between MDD-W/HFIAS and BMI be interpreted with caution; we suggest that biochemical (e.g., hemoglobin, ferritin, vitamin A, B12) and functional measures (e.g., grip strength) be explored in future work.

Our findings align with regional studies reporting high food insecurity among women with HIV in SSA [28], as well as with studies linking depression to poorer nutritional outcomes [4]. The observed MDD-W mean (~4.3) and low egg/dairy consumption mirror nutritional patterns reported in similar low-resource urban settings [29]. Where such studies have found associations between food insecurity and BMI or ART outcomes, differences in study design, measurement, or context (rural vs. urban, availability of food assistance) may explain the divergence in results [27,30,31].

Mental health screening and integrated psychosocial support should be considered essential components of HIV care packages. Given the independent association between probable depression and lower BMI, addressing depressive symptoms may help improve nutritional outcomes; however, this hypothesis would require longitudinal and interventional confirmation of the impact this intervention could have on overall well-being. The high prevalence of food insecurity and inadequate dietary diversity indicates a need for context-appropriate nutrition interventions in the form of both food rations and measures to improve dietary quality (e.g., vouchers, livelihood programs, agricultural support, micronutrient supplementation, and behavior-change communication focused on affordable, nutrient-dense foods). Nutrition programs should not solely rely on BMI as an outcome, but incorporate additional functional and micronutrient indicators, dietary quality metrics, and measures of food access and stability.

### 4.1. Strengths and Limitations

In our cross-sectional analysis of 571 women living with HIV and receiving antiretroviral therapy (ART) in Kinshasa, we acknowledge several limitations that may affect the interpretation of our findings. Firstly, the cross-sectional design inherently restricts our ability to establish causal relationships between variables, such as the associations observed between probable depression, food insecurity, dietary diversity, and body mass index (BMI). The retrospective nature of the baseline BMI data also raises concerns about reverse causation, as the temporal sequence between exposures and outcomes cannot be firmly established.

Moreover, while we utilized validated instruments such as the Household Food Insecurity Access Scale (HFIAS) and the Minimal Dietary Diversity-Women (MDD-W), the reliance on self-reported adherence to ART and 24-h dietary recall methods may introduce recall and social desirability biases. These biases could undermine the relationships we observed, as participants might have over- or under-reported their dietary intake and medication adherence. The assessment of dietary diversity was limited to a single 24-h period, which may not accurately reflect habitual consumption patterns or absolute nutrient intakes. Additionally, BMI, as a metric, is not exhaustive; it does not provide comprehensive insight into micronutrient deficiencies or body composition, particularly relevant in populations living with HIV. Our findings indicate that while BMI remains a common measure, it may not adequately capture the complexities of nutritional status, especially when individuals can have a normal BMI yet still experience micronutrient insufficiencies.

Furthermore, the study sample was derived exclusively from urban clinical settings in Kinshasa, which may limit the generalizability of our findings to rural or non-clinic populations. Seasonal variations in food availability were also not accounted for, which could impact on our measurements of dietary diversity and food insecurity. In light of these limitations, we recommend that future research includes longitudinal designs with repeated dietary assessments and objective measures of nutritional status to clarify the causal pathways between mental health, food security, and nutritional outcomes among women living with HIV. Incorporating biochemical evaluations and functional measures would provide a more nuanced understanding of the participants’ nutritional health.

### 4.2. Policy and Programmatic Recommendations

We recommend integrating routine depression screening (e.g., brief validated tools) and accessible mental health services within ART clinics, as well as piloting co-located psychosocial interventions to evaluate nutritional impact and ART outcomes. We also suggest designing nutrition-sensitive interventions that prioritize dietary diversity (e.g., vouchers for nutrient-rich foods and promoting home gardens and community kitchens), in addition to energy supplementation where needed. We recommend that monitoring be strengthened by incorporating assessments of dietary quality, food security, and micronutrient status into ART program evaluation. We also recommend considering targeted support for women newly initiated on ART, particularly during the 3–6-year window when BMI gains appear largest; this may be an optimal period for consolidating nutritional recovery.

### 4.3. Suggestions for Future Research

We suggest that prospective cohort or intervention studies be used to clarify causal pathways between depression, food insecurity, dietary diversity, and long-term anthropometric and clinical outcomes (viral suppression, morbidity, and mortality). We also recommend the use of objective adherence measures (pharmacy refill data and electronic pill boxes) and repeated dietary assessments across seasons to reduce measurement error. Further studies should examine body composition and micronutrient status (e.g., hemoglobin, vitamin A, vitamin B12, zinc, serum ferritin) to detect clinically relevant nutritional deficits not captured by BMI. Future research should also evaluate the cost-effectiveness and sustainability of integrated mental health–nutrition packages within ART programs in urban Congolese settings using a validated mental health diagnostic tool. Randomized or quasi-experimental evaluations of integrated packages—combining mental health counseling, nutrition education, and targeted food assistance—could identify the benefits and cost-effectiveness of programmatic integration within ART services.

## 5. Conclusions

In this sample of women on ART attending clinics in Kinshasa, probable depressive symptoms were associated with a modestly lower BMI after adjusting for the baseline BMI and other covariates. Household food insecurity and dietary diversity were common but not independently associated with BMI in the adjusted analyses; this result may reflect the limitations of BMI and single-day dietary assessments. Future longitudinal and interventional studies should include repeated dietary measures, viral suppression/ART regimen data, and objective nutritional biomarkers and functional outcomes to clarify causal pathways and inform integrated mental health and nutrition interventions. Programmatic recommendations to integrate mental health screening into HIV care should be considered hypothesis-generating until tested in longitudinal or experimental designs.

## Figures and Tables

**Table 1:** Patients’ sociodemographic characteristics.

	*n*	%
Age (mean ± SD)	42.3 ± 12.5	
Age groups		
18–24	55	9.6
25–34	111	19.4
35–49	225	39.4
50+	180	31.5
Level of education attained		
None/primary	111	19.4
Secondary	315	55.2
University	145	25.4
Marital status		
Never married	178	31.2
Divorced/widowed/separated	191	33.5
Common-law/polygamous marriage	66	11.6
Monogamous marriage	136	23.8
Household size		
Less than 5	232	40.7
Six and above	338	59.3
Household wealth tertile		
Lowest tertile	244	42.8
Middle tertile	136	23.9
Highest tertile	190	33.3
Year since induction of ARV (median (Q1–Q3))	3.99 (1.61–7.12)	
Alcohol consumption *		
No	470	82.5
Yes	100	17.5
Tobacco consumption *		
No	529	93.1
Yes	39	6.9
Total	571	100

*: 1 to 3 missing data points.

**Table 2:** Study participants’ food insecurity access-related conditions.

	Households Experienced the Condition at Any Time During the Recall Period (%)		Households Often Experienced the Condition (%)	
	*n* *	% *	*n* *	%
Experienced food insecurity access-related conditions at any time during the recall period (%)				
Worry that her household would not have enough food	420	73.9	0	0.0
Unable to eat the kinds of foods they preferred because of a lack of resources	420	73.7	0	0.0
Eat a limited variety of foods due to a lack of resources	409	71.8	15	3.7
Eat some foods that they really did not want to eat because of a lack of resources	416	73	12	2.9
Eat a smaller meal than she felt she needed because there was not enough food	404	70.9	14	3.5
Eat fewer meals in a day because there was not enough food	400	70.2	4	1.0
No food of any kind to eat in the household	398	69.8	4	1.0
Went to sleep at night hungry because there was not enough food	383	67.2	3	0.8
Went a whole day and night without eating anything because there was not enough food	378	66.3	1	0.3

* Percentage calculated for *N* = 570; *n* is the number of respondents who experienced the condition.

**Table 3:** Consumption frequency of each food group.

	*n* *	% *
Grains, white roots and tubers, and plantains	558	97.7
Pulses (beans, peas and lentils)	540	94.6
Nuts and seeds	182	31.9
Dairy	173	30.3
Meat, poultry and fish	392	68.7
Eggs	50	8.8
Dark green leafy vegetables	380	66.5
Other vitamin A-rich fruits and vegetables	132	23.1
Other vegetables	509	89.1
Other fruits	182	31.9
MDD_W (Mean ± SD)	4.27 ± 1.31	
Dietary diversity		
Inadequate diversity	329	57.6
Adequate diversity	242	42.4

* Percentage calculated for *N* = 571; *n* is the number of respondents who consumed each food group.

**Table 4:** Linear regression predicting BMI at follow-up from baseline BMI *.

BMI at Survey	Unstandardized β	Standardized β	St. Err	*t*-Value	*p*-Value	95% CI	
Centered baseline BMI	0.479	2.423	0.053	9.00	<0.001	0.375	0.585
ART adherence							
Adherent vs. non-adherent	−0.034	−0.016	0.353	−0.10	0.923	−0.727	0.659
Depression symptoms							
Presence vs. absence	−0.987	−0.490	0.373	−2.65	0.008	−1.720	−0.255
Food security							
Food-insecure vs. food-secure	−0.524	−0.230	0.396	−1.32	0.186	−1.302	0.254
Dietary diversity score	0.162	0.210	0.135	1.21	0.228	−0.102	0.427
Centered time	0.100	0.427	0.047	2.13	0.034	0.008	0.193
Women’s age	0.010	0.129	0.013	0.79	0.427	−0.015	0.036
Wealth index							
Middle vs. first tertile	−0.119	−0.051	0.409	−0.29	0.769	−0.923	0.683
Highest vs. first tertile	0.519	0.245	0.430	1.21	0.228	−0.325	1.363
Constant	22.953	-	0.931	24.64	<0.001	21.123	24.783
Mean dependent var		23.384			SD dependent var		4.432
R-squared		0.354			Number of obs.		503
F-test		16.020			Prob > F		<0.001
Akaike crit. (AIC)		2724.014			Bayesian crit. (BIC)		2766.220

* Estimates are from ANCOVA adjusting for baseline BMI and covariates, with robust standard errors.

## Data Availability

A de-identified dataset and codebook are available at OSF (DOI: 10.17605/OSF.IO/35NJX). Any additional materials that cannot be shared openly due to ethical restrictions are available from KSPH upon reasonable request.

## Supplementary Materials

The following supporting information can be downloaded at: https://www.mdpi.com/article/10.3390/nu17203230/s1, Figure S1: Histogram of BMI distribution; Figure S2: Changes in BMI, both overall and by key exposures; Figure S3: The Relationship Between Dietary Diversity Score, Age, Baseline BMI, Time Since Induction of ART, and Follow-up BMI; Figure S4: Model diagnostics: residuals-versus-fitted and Q–Q plots; Table S1: Frequency of missing data by key variables; Table S2: Baseline and follow-up BMI of main covariates; Table S3: BMI categories baseline and survey by key exposures; Table S4: Linear regression predicting BMI at follow-up from baseline BMI (all participants vs. 15-49 age group); Table S5: Regression analyses on BMI follow-up: linear vs. mixed-effects models with key associated factors; Table S6: Comparative analysis of BMI follow-up estimations: complete case versus multiple imputation approaches.

## Abbreviations

ANCOVA	Analysis of covariance
ART	Antiretroviral therapy
BMI	Body mass index
CI	Confidence interval
DRC	Democratic Republic of Congo
FANTA	Food and Nutrition Technical Assistance
HFIAS	Household Food Insecurity Access Scale
HIV	Human immunodeficiency virus
HSCL-10	Hopkins Symptom Checklist-10
MDD_W	Minimum Dietary Diversity for Women
MICE	Multiple imputation by chained equations
PLHIV	People living with HIV
SEP	Socioeconomic position
SSA	Sub-Saharan Africa
USAID	United States Agency for International Development
VIFs	Variance inflation factors
WHO	World Health Organization
WLHIV	Women living with HIV
