# Supplementary material for: Probable Depression Is Associated with Lower BMI Among Women on ART in Kinshasa, the Democratic Republic of Congo: A Cross-Sectional Study
Source: Nutrients. 2025 Oct 15;17(20):3230. doi: 10.3390/nu17203230 (PMC12567450; doi:10.3390/nu17203230)
Supplement: Supplementary file 1 [file nutrients-17-03230-s001.zip › Supplemental tables_1.pdf]

nutrients MDPI

Table S1. Frequency of missing data by key variables.

Variable	n	%
Age	0	0.0
Depression scores	0	0.0
Food security	1	0.2
Dietary diversity	0	0.0
Wealth index	1	0.2
Adherence	3	0.5
Follow-up BMI	13	2.3
Baseline BMI	32	5.6

Table S2. Baseline and follow-up BMI of main covariates.

Baseline BMI (Kg/m^2)			Follow-up BMI (Kg/m^2)		
Depressive	Non-depressive	*p*	Depressive	Non-depressive	*p*
19.68 ± 5.68	21.37 ± 4.39	0.0001	22.36 ± 4.11	24.14 ± 4.45	<0.001
Food secure	Food insecure	*p*	Food secure	Food insecure	*p*
20.56 ± 5.56	20.64 ±4.87	0.878	23.51 ± 4.16	23.33 ±4.47	0.682
Adequate diversity	Inadequate diversity	*p*	Adequate diversity	Inadequate diversity	*p*
20.59± 5.46	20.67 ± 4.74	0.856	23.59± 4.15	23.25 ± 4.56	0.375
Adherent to ART	Non-adherent to ART	*p*	Adherent to ART	Non-adherent to ART	*p*
20.84 ± 4.70	20.28 ± 5.75	0.233	23.49 ± 4.38	23.21 ± 4.44	0.486

Table S3. BMI categories baseline and survey by key exposures.

	Baseline BMI									Follow-up BMI								
	Underweight		Normal		Overweight		Obese			Underweight		Normal		Overweight		Obese		
	n	%	n	%	n	%	n	%	*p*	n	%	n	%	n	%	n	%	*p*
**Adherence to ART**									0.397									0.441
Non-adherent	55	32.2	87	50.9	24	14.0	5	2.9		23	13.1	102	58.0	38	21.6	13	7.4	
Adherent	97	26.6	213	58.4	43	11.8	12	3.3		33	8.7	236	62.3	84	22.2	26	6.9	
Total	152	28.4	300	56.0	67	12.5	17	3.2		56	10.1	338	60.9	122	22.0	39	7.0	
**Depressive symptoms**									0.013									<0.001
Non-depressive	71	23.3	180	59.0	43	14.1	11	3.6		20	6.2	191	59.3	79	24.5	32	9.9	
Depressive	84	35.9	120	51.3	24	10.3	6	2.6		36	15.3	149	63.1	44	18.6	7	3.0	
Total	155	28.8	300	55.7	67	12.4	17	3.2		56	10.0	340	60.9	123	22.0	39	7.0	
**Food insecurity category (access)**									0.806									0.420
Food secure	41	29.5	74	53.2	20	14.4	4	2.9		11	7.9	87	62.1	35	25.0	7	5.0	
Food insecure	114	28.6	226	56.6	46	11.5	13	3.3		45	10.8	253	60.7	87	20.9	32	7.7	
Total	155	28.8	300	55.8	66	12.3	17	3.2		56	10.1	340	61.0	122	21.9	39	7.0	
**Minimum dietary diversity for women**									0.317									0.004
Inadequate	97	31.3	168	54.2	34	11.0	11	3.5		44	13.7	181	56.2	74	23.0	23	7.1	
Adequate	58	25.3	132	57.6	33	14.4	6	2.6		12	5.1	159	67.4	49	20.8	16	6.8	
Total	155	28.8	300	55.7	67	12.4	17	3.2		56	10.0	340	60.9	123	22.0	39	7.0	

Table S4. Linear regression predicting BMI at follow-up from baseline BMI (all participants vs. 15-49 age group).

BMI at Survey	All participants					15-49 group			
	Unstandardized β	Standardized β	*p*-Value	95% CI*		Unstandardized β	Standardized β	95% CI*	
Centered baseline BMI	0.479	2.423	<0.001	0.375	0.585	0.473	2.329	0.334	0.611
Adherence to ART									
Adherent vs. non-adherent	−0.034	-0.016	0.923	−0.727	0.659	0.182	0.086	-0.657	1.022
Depression symptoms									
Presence vs. absence	−0.987	-0.490	0.008	−1.72	−0.255	-0.882	-0.440	-1.753	-0.011
Food security									
Food insecure vs. food secure	−0.524	-0.230	0.186	−1.302	0.254	-0.487	-0.215	-1.407	0.432
Dietary diversity score	0.162	0.210	0.228	−0.102	0.427	0.184	0.228	-.1677	0.534
Centered time	0.100	0.427	0.034	0.008	0.193	0.154	0.558	0.009	0.299
Women's age	0.010	0.129	0.427	−0.015	0.036	0.039	0.346	-0.005	0.083
Wealth index									
Middle vs. first tertile	−0.119	-0.051	0.769	−0.923	0.683	-0.018	-0.008	-0.909	0.872
Highest vs. first tertile	0.519	0.245	0.228	−0.325	1.363	0.433	0.198	-0.627	1.493
Constant	22.953	-	<0.001	21.123	24.783	21.75		19.27	24.227
Mean dependent var	23.38		SD		4.432	Mean dependent var	23.34	SD	4.30
R-squared	0.356		Number of obs.		503	R-squared	0.36	Number of obs.	342

*95%CI for unstandardized β.

Table S5. Regression analyses on BMI follow-up: linear vs. mixed-effects models with key associated factors.

	ANCOVA	Multilevel (mixed-effects) model
Variable	bmi_followup	bmi_followup
Centered baseline BMI	0.480***	0.480***
	(0.0533)	(0.0528)
Adherence (adherent vs. non-adherent)	-0.0342	-0.0342
	(0.353)	(0.350)
Depression symptoms (presence vs. absence)	-0.987***	-0.987***
	(0.373)	(0.370)
Food security (food insecure vs. food secure)	-0.524	-0.524
	(0.396)	(0.392)
Dietary diversity score	0.162	0.162
	(0.135)	(0.133)
Centered time	0.100**	0.100**
	(0.0473)	(0.0468)
Women's age	0.0103	0.0103
	(0.0129)	(0.0128)
Wealth index (middle vs. first tertile)	-0.120	-0.120
	(0.409)	(0.405)
Wealth index (highest vs. first tertile)	0.519	0.519
	(0.430)	(0.426)
Constant	22.95***	22.95***
	(0.931)	(0.923)
Observations	503	503

Standard errors in parentheses; *** $p<0.01$.

Estimates shown as mean, with robust standard errors.

Table S6. Comparative analysis of BMI follow-up estimations: complete case versus multiple imputation approaches.

	Complete Case Analysis					MICE				
BMI at Survey	Unstandardized β	St. Err*	*p*-Value	95% CI		Unstandardized β	St. Err*	*p*-Value	95% CI	
Baseline BMI	0.471	0.0322	<0.001	0.407	0.533	0.462	0.0309	<0.001	0.402	0.524
Adherence to ART										
Adherent vs. non-adherent	-0.0504	0.341	0,882	−0.720	0.619	-0.0450	0.333	0.922	-0.699	0.61
Depression symptoms										
Presence vs. absence	-1.134	0.358	0.002	−1.836	−0.431	-1.238	0.35	<0.001	-1.926	-0.550
Food security										
Food insecure vs. food secure	-0.579	0.385	0,133	−1.335	0.177	-0.605	0.379	0.111	-1.350	0.139
Dietary diversity score	0.117	0.124	0,348	−0.127	0.361	0.121	0.118	0.307	-1.112	0.354
Time since ART initiation	0.0169	0.013	0,177	−0.008	0.041	0.015	0.0125	0.217	-1.009	0.040
Women's age	0.0207	0.0130	0,112	−0.005	0.046	0.018	0.0126	0.141	-1.006	0.044
Wealth index										
Middle vs. first tertile	0.0193	0.418	0,963	−0.801	0.839	0.010	0.4058	0.980	-0.787	0.807
Highest vs. first tertile	0.611	0.380	0,108	−0.135	1.357	0.582	0.372	0.118	-0.149	1.313
Constant	12.94	1.158	<0.001	10.662	15.214	13.24	1.115	<0.001	11.054	15.437

MICE: multiple imputations by chained equations. *: we did not utilize robust standard errors.
