# Supplementary figures and images for: Probable Depression Is Associated with Lower BMI Among Women on ART in Kinshasa, the Democratic Republic of Congo: A Cross-Sectional Study
Source: Nutrients. 2025 Oct 15;17(20):3230. doi: 10.3390/nu17203230 (PMC12567450; doi:10.3390/nu17203230)

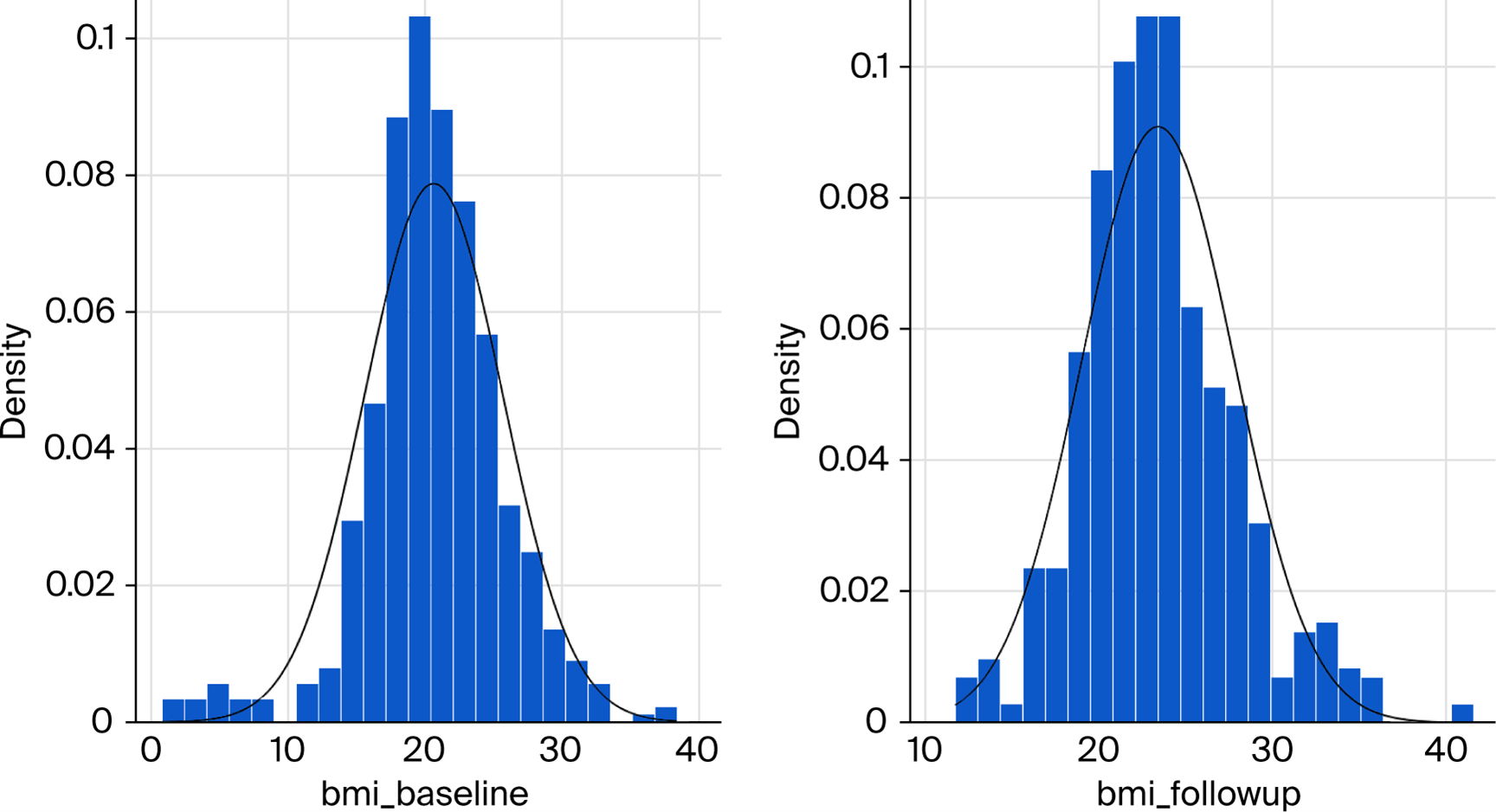

Supplement: Supplementary file 1 [file nutrients-17-03230-s001.zip › Figure S1.png]

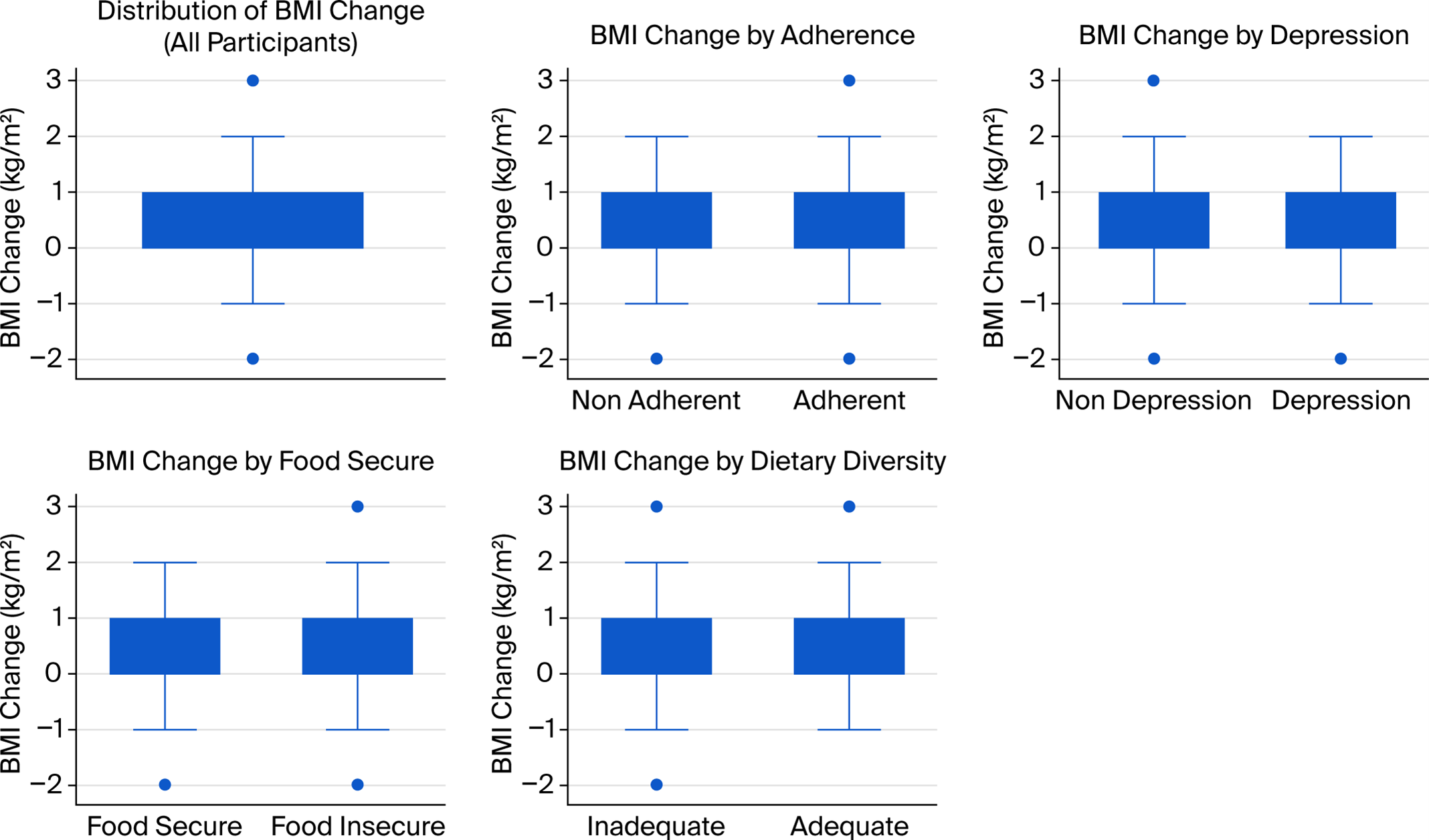

Supplement: Supplementary file 1 [file nutrients-17-03230-s001.zip › Figure S2.png]

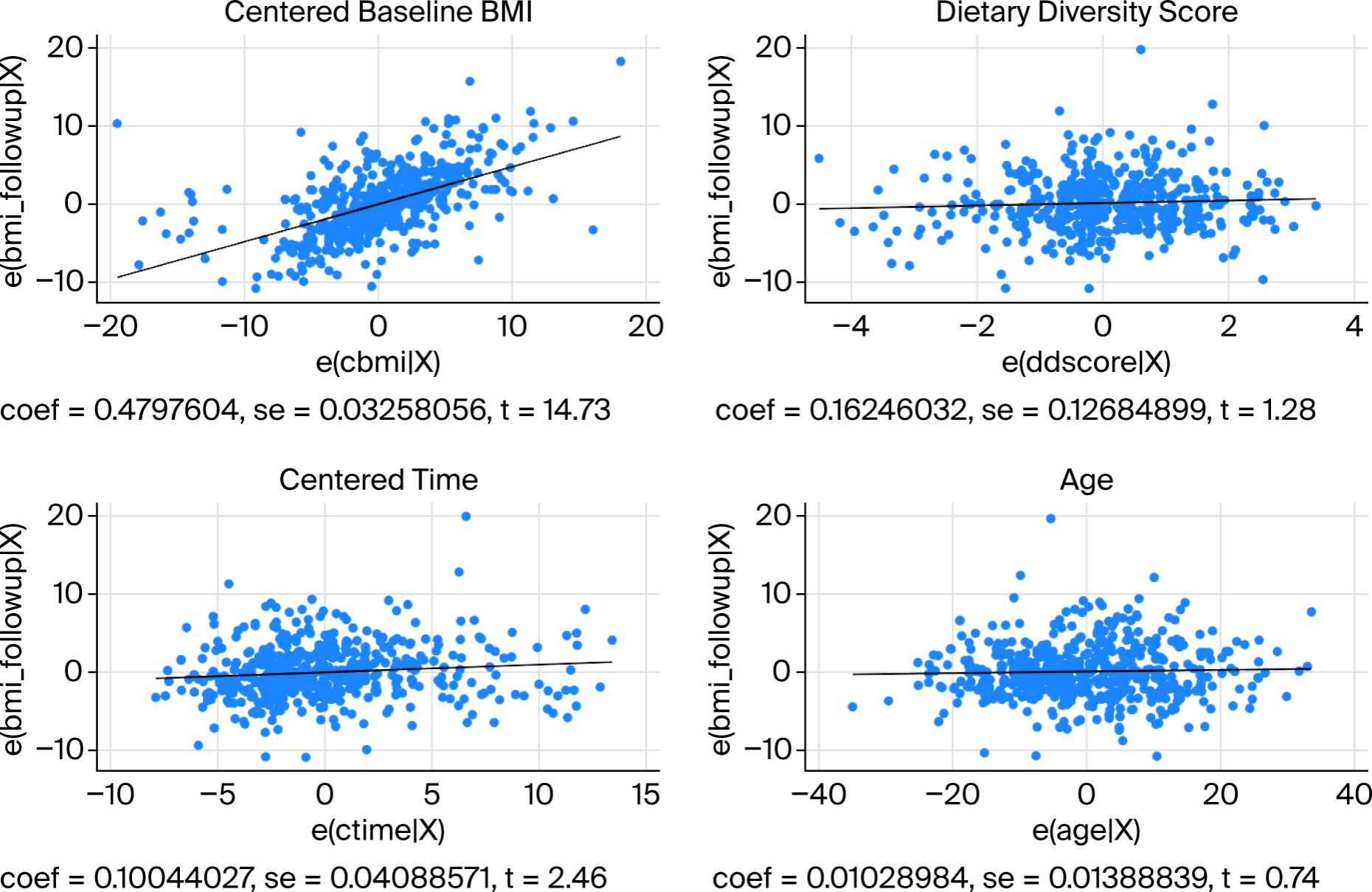

Supplement: Supplementary file 1 [file nutrients-17-03230-s001.zip › Figure S3.png]

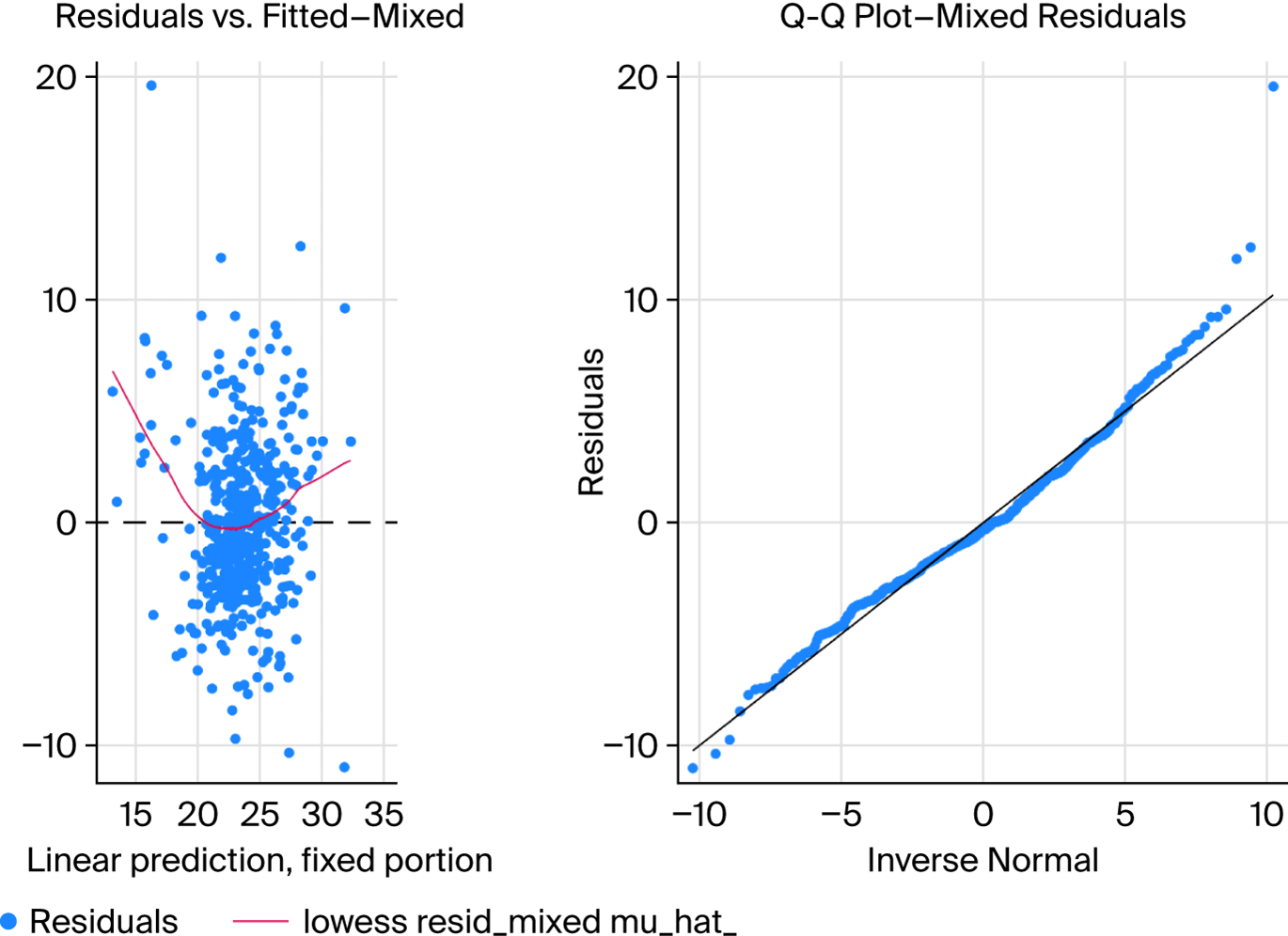

Supplement: Supplementary file 1 [file nutrients-17-03230-s001.zip › Figure S4.png]
